# Supplementary material for: Topological links and knots of speckled light mediated by coherence singularities
Source: Light Sci Appl. 2025 Apr 27;14:175. doi: 10.1038/s41377-025-01865-3 (PMC12034767; doi:10.1038/s41377-025-01865-3)
Supplement: Supplementary file 1 — Supplementary information for Topological links and knots of speckled light mediated by coherence singularities [file 41377_2025_1865_MOESM1_ESM.docx]

**Supplementary Information for:**

**Topological links and knots** **of speckled light mediated by coherence singularities**

Zhuoyi Wang^1,5^, Xingyuan Lu^1,5^, Zhigang Chen^2🖂^, Yangjian Cai^3,4🖂^ and Chengliang Zhao^1🖂^

^1^School of Physical Science and Technology, Jiangsu Key Laboratory of Frontier Material Physics and Devices & Suzhou Key Laboratory of Intelligent Photoelectric Perception, Soochow University, Suzhou 215006, China

^2^The MOE Key Laboratory of Weak-Light Nonlinear Photonics*,* TEDA Applied Physics Institute and School of Physics, Nankai University, Tianjin 300457, China.

^3^Shandong Provincial Engineering and Technical Center of Light Manipulations & Shandong Provincial Key Laboratory of Optics and Photonic Device, School of Physics and Electronics, Shandong Normal University, Jinan 250358, China

^4^Joint Research Center of Light Manipulation Science and Photonic Integrated Chip, East China Normal University, Shanghai 200241, China

^5^These authors contributed equally

🖂e-mail: zgchen@nankai.edu.cn (Z. Chen); yangjiancai@sdnu.edu.cn (Y. Cai); zhaochengliang@suda.edu.cn (C. Zhao)

**Supplementary Note 1: Analysis of coherence singularities and incoherent topological structures composed of speckles**

In Fig. S11, a linearly polarized beam with a wavelength of λ = 532 nm passes through L1 and the scattered object, whose electric field is expressed as:

$E\left( \mathbf{r} \right)\mathbf{=}A_{0}\exp\left( -\frac{\mathbf{r}^{2}}{2\sigma_{0}^{2}} \right)\tau\left( \mathbf{r},t \right)$ (S1)

where $A_{0}$ is a constant that has the dimension of an optical intensity, and $\sigma_{0}$ indicates the beam waist. After the phase becomes random due to the non-uniform distribution of the scattered object. $\tau\left( \mathbf{r},t \right)=a_{n}\left( \mathbf{r},t \right)+ib_{n}\left( \mathbf{r},t \right)$ where $a_{n}$ and $b_{n}$ denote random functions that are independent of each other. As the scattered object rotates, the electric field after passing through the thin lens L2 with focal length *f*_2_ is given in ref. [1]:

$T\left( \boldsymbol{\rho},t \right)=FFT\left[ \exp\left( -\frac{\mathbf{r}^{2}}{2\sigma_{0}^{2}} \right)\tau\left( \mathbf{r},t \right) \right]$ (S2)

where *FFT* denotes the Fourier transform. $\mathbf{r}$ and $\boldsymbol{\rho}$ denote the coordinates of the different planes.

A periodic N-strand braid can be represented by the polynomials $q\left( u,v \right)=u^{2}-v^{n}$, where *q* is the composite function that forms the links and knots. *u* and *v* are functions related to spatial coordinates (**r**, *z*), written as $u=\frac{\left[ \left( \mathbf{r}^{2}+z^{2}-1 \right)+2iz \right]}{\mathbf{r}^{2}+z^{2}+1}$, and $v=\frac{2\left( x+iy \right)}{\mathbf{r}^{2}+z^{2}+1}$, where **r** = (*x*, *y*) [2]. Then, two kinds of linked loops, Hopf link for *n*=2 and a Trefoil knot for *n*=3, are used. Applying this concept to the optical field, Berry et al [3] proposed the use of vortex phase singularities to construct a 3D topological structure that benefits from the diffraction of the electric field. In fully coherent experiments, LG beams can be coherent-superimposed to generate a specific beam under paraxial propagation conditions. The coefficients in the coherent superposition of the LG function are designed to realize perturbations, which is realized via iterative optimization [4]. Then, the hologram for generating optical links and knots can be designed as

$H\left( \boldsymbol{\rho} \right)=H_{per}\left( \boldsymbol{\rho} \right)+H_{unper}\left( \boldsymbol{\rho} \right)=\sum C_{n}{LG}_{p}^{l}\left( \boldsymbol{\rho} \right)$ (S3)

Here, $H_{per}\left( \boldsymbol{\rho} \right)$ is the perturbed wave used to separate the singularities, and $H_{unper}\left( \boldsymbol{\rho} \right)$is the vortex term with topological charge *l*. ${LG}_{p}^{l}$ denotes the Laguerre-Gauss beam, *l* is the topological charge, and *p* is the radial index. The optimized coefficients $C_{n}$ used in this work are listed in Table S1 based on the theoretical model proposed by Dennis [4]. After passing through the hologram, the instantaneous electric field can be specified as:

$E\left( \boldsymbol{\rho},t \right)\mathbf{=}H\left( \boldsymbol{\rho} \right)T\left( \boldsymbol{\rho},t \right)$ (S4)

Then, the cross-spectral density changes to $W\left( \boldsymbol{\rho}_{1}\boldsymbol{,\rho}_{2} \right)=\left\langle E^{*}\left( \boldsymbol{\rho}_{1} \right)E\left( \boldsymbol{\rho}_{2} \right) \right\rangle$. The diffraction order +1 modulation function of the SLM is $H\left( \boldsymbol{\rho} \right)=A\left( \boldsymbol{\rho} \right)\exp\left[ i\varphi\left( \boldsymbol{\rho} \right) \right]$, after passing through the SLM, the electric field can be expressed as:

$E\left( \boldsymbol{\rho},t \right)\mathbf{=}A\left( \boldsymbol{\rho} \right)\exp\left[ i\varphi\left( \boldsymbol{\rho} \right) \right]T\left( \boldsymbol{\rho},t \right)$ (S5)

Generally, the statistical behavior of $T\left( \boldsymbol{\rho},t \right)$decides the coherence of the incoherent light field, and the statistical properties of a long period of time as the dynamic scatterer rotates can be described by the cross-spectral density function [1]:

$$W\left( \boldsymbol{\rho}_{1}\boldsymbol{,\rho}_{2} \right)=\left\langle E^{*}\left( \boldsymbol{\rho}_{1} \right)E\left( \boldsymbol{\rho}_{2} \right) \right\rangle$$

=$H^{\boldsymbol{*}}\left( \boldsymbol{\rho}_{1} \right)H\left( \boldsymbol{\rho}_{2} \right)\left\langle T^{\boldsymbol{*}}\left( \boldsymbol{\rho}_{1} \right)T\left( \boldsymbol{\rho}_{2} \right) \right\rangle$

=$H^{\boldsymbol{*}}\left( \boldsymbol{\rho}_{1} \right)H\left( \boldsymbol{\rho}_{2} \right)\exp\left[ -\frac{\left| \boldsymbol{\rho}_{1}-\boldsymbol{\rho}_{2} \right|^{2}}{2\delta_{0}^{2}} \right]$ (S6)

The asterisk denotes the complex conjugate and the angular brackets denote the ensemble average. $\delta_{0}={\lambda f_{2}}/{\pi\sigma_{0}}$ represents the transverse coherence width of the incoherent beam. After a transmission distance *z*, the cross spectral density function becomes:

$W\left( \mathbf{r}_{1}\mathbf{,r}_{2},z \right)=\left( \frac{k}{2\pi z} \right)^{2}\iint H^{\boldsymbol{*}}\left( \boldsymbol{\rho}_{1} \right)H\left( \boldsymbol{\rho}_{2} \right)\exp\left[ -\frac{\left| \boldsymbol{\rho}_{1}-\boldsymbol{\rho}_{2} \right|^{2}}{2\delta_{0}^{2}} \right]H^{\boldsymbol{*}}\left( \boldsymbol{\rho}_{1} \right)H\left( \boldsymbol{\rho}_{2} \right)$

$\times\exp\left[ -\frac{ik}{2z}\left( \mathbf{r}_{1}-\boldsymbol{\rho}_{1} \right)^{2}+\frac{ik}{2z}\left( \mathbf{r}_{2}-\boldsymbol{\rho}_{2} \right)^{2} \right]d\boldsymbol{\rho}_{1}d\boldsymbol{\rho}_{2}$ (S7)

The coherence function at transmission distance *z* is written as:

$\mu\left( \mathbf{r}_{1}\mathbf{,r}_{2},z \right)=\frac{W\left( \mathbf{r}_{1}\mathbf{,r}_{2},z \right)}{\sqrt{W\left( \mathbf{r}_{1}\mathbf{,r}_{1},z \right)}\sqrt{W\left( \mathbf{r}_{2}\mathbf{,r}_{2},z \right)}}$ (S8)

Singularities in the described incoherent light field, are called coherence singularities. Such singularities are pairs of points with zero degree of coherence, that is

$\left| \mu\left( \mathbf{r}_{1}\mathbf{,r}_{2},z \right) \right|=0$ (S9)

The incoherent links and knots are constructed by linking these coherence singularities in different planes.

**Supplementary Note 2: Experimental scheme for measurement of incoherent properties and topological structures**

As shown in Fig. S11, the measurement was conducted at the back focus plane of L4 (z=0, indicated by a dashed line in Supplementary Fig. S11). Altering the placement of the CCD at various transmission distances facilitated the acquisition of intensity data at varying propagation distances (Fig. S11b). The coherence structure was reconstructed from diffraction intensities at the source plane utilizing a multi-probe ptychography iterative algorithm. Another scattering object (USAF) was positioned perpendicular to the optical axis, and the diffraction intensity was captured by an EMCCD camera (iXon Life, Oxford). In order to obtain sufficient diffraction data, a two-dimensional mobile stage (CONEX-MFACC Newport) was utilized to perform overlapping scans of the object. Subsequently, the incoherent beam was comprehensively characterized, encompassing both intensity and coherence structure. The distance between the USAF and EMCCD was measured at 146 mm. A total of 400 raw diffraction patterns were acquired through 20 × 20 overlapping scans with a step size of 40*μ*m. In accordance with incoherent superposition theory, the coherence structure can be quantitatively measured.

As an incoherent light field, the cross spectral density can be regarded as the incoherent superposition of multiple electric fields, that is $W\left( \boldsymbol{\rho}_{1},\boldsymbol{\rho}_{2} \right)=\sum_{n} E_{n}(\boldsymbol{\rho}_{1})E_{n}^{*}\left( \boldsymbol{\rho}_{2} \right)$. On the camera plane, each recorded intensity $I_{0} (\mathbf{k})$ can also be treated as the incoherent superposition of diffraction patterns for different modes, that is $I_{0} (\mathbf{k})=\sum_{n} \psi_{n}(\mathbf{k})\psi_{n}^{*}\left( \mathbf{k} \right)$. Then, the modes on the object plane can be updated using the multi-probe ptychography iterative engine [5]. The purpose of the multi-probe ptychography iterative engine is to minimize the difference between the collected intensity and the calculated average-intensity. For each mode on the camera plane $\psi_{n}$($\mathbf{k}$) and object plane $E_{n}$($\boldsymbol{\rho}$), according to the gradient descent algorithm, we can update the camera plane $\psi_{n}$($\mathbf{k}$) and object plane $E_{n}$($\boldsymbol{\rho}$) can be respectively updated as:

$$\psi_{n}^{i^{'}}\left( \mathbf{k} \right)=\frac{\sqrt{I_{0}\left( \mathbf{k} \right)}}{\sqrt{\sum_{n=1}^{N} \psi_{n}^{i}\left( \mathbf{k} \right)\psi_{n}^{*i}\left( \mathbf{k} \right)}}\psi_{n}^{i}\left( \mathbf{k} \right)$$

$E_{n}^{i+1}\left( \boldsymbol{\rho} \right)=E_{n}^{i}\left( \boldsymbol{\rho} \right)+\beta\frac{O^{*}\left( \boldsymbol{\rho} \right)}{\left| O\left( \boldsymbol{\rho} \right) \right|_{max}^{2}}\left[ {\Phi^{'}}_{n}^{i}\left( \boldsymbol{\rho} \right)-\Phi_{n}^{i}\left( \boldsymbol{\rho} \right) \right]$ (S10)

where ${\Phi^{'}}_{n}^{i}\left( \boldsymbol{\rho} \right)=\mathcal{F}^{-1}\left\{ \psi_{n}^{i^{'}}\left( \mathbf{k} \right) \right\}$. 𝛒 is the coordinate on the object plane and **k** is the coordinate on the detector plane. $I_{0}\left( \mathbf{k} \right)$ represents the experimentally recorded diffraction intensity. $\psi_{n}^{i'}(\mathbf{k})$is updateable complex diffraction field. “*n*” means the *n*-th mode, whose total number is *N*. “*i*” is the *i-*th iteration. $\beta$ is an update factor, which was set to 0.9 in this work. Based on Eq. (S10), $E_{n}$($\boldsymbol{\rho}$) was reconstructed after 200 iterations. After a set of light modes $\left\{ E_{n}\left( \boldsymbol{\rho} \right) \right\}$ was reconstructed, the cross-spectral density was calculated based on $W\left( \boldsymbol{\rho}_{1}\mathbf{,}\boldsymbol{\rho}_{2} \right)=\sum_{n=1}^{N} E_{n}^{*}\left( \boldsymbol{\rho}_{1} \right)E_{n}\left( \boldsymbol{\rho}_{2} \right)$[5], and the coherence structure was calculated via Eq. (2). The unique advantage that distinguishes the incoherent decomposition method from two-dimensional measurement schemes is the complete four-dimensional information acquisition which ensures the calculation of second-order statistics on other planes based on angular spectrum propagation (see Fig. 3b). The reconstructed pixel size satisfies the formula: $\Delta x=\lambda z/L$ and the ideal resolution is approximately two times the pixel size. z represents the distance from the scattering object to the camera. *L* represents the camera size. Therefore, the larger the camera size, the higher the resolution.

Within the accuracy of paraxial approximation, the incoherent light field can be calculated by mode superposition and angular spectral diffraction when it is transmitted in free space. By applying the transmission formula discussed in Supplementary Note 1 S7, the propagation formula can be expressed in the following alternative form

$W\left( \mathbf{r}_{1}\mathbf{,}\mathbf{r}_{2} \right)=\sum_{n=1}^{N} \mathcal{F}\left\{ E_{n}^{*}\left( \boldsymbol{\rho}_{1} \right) \right\}\mathcal{F}\left\{ E_{n}\left( \boldsymbol{\rho}_{2} \right) \right\}$ (S10)

Here $\mathcal{F}$ is a function describing that the beam is transmitted from the $\boldsymbol{\rho}$ plane to the **r** plane using angular spectrum theory[6].

**Table S1. The coefficients** $\boldsymbol{C}_{\boldsymbol{n}}$ **for** $\boldsymbol{LG}_{\boldsymbol{p}}^{\boldsymbol{l}}$ **with different *p* and *l*.**

|  |  | Hopf link | Trefoil knot |
| --- | --- | --- | --- |
| *l* | *p* | $C_{n}$ | $C_{n}$ |
| 0 | 0 | 2.63 | 1.51 |
| 0 | 1 | -11.04 | -6.34 |
| 0 | 2 | 26.52 | 15.22 |
| 0 | 3 | -27.38 | -15.72 |
| 0 | 4 | 12.59 | 7.23 |
| 0 | 5 | -2.63 | -1.51 |
| 2 | 0 | -10.65 | 0 |
| 3 | 0 | 0 | -6.06 |

**References**

[1] Tong R. et al. Fast calculation of tightly focused random electromagnetic beams: controlling the focal field by spatial coherence. *Opt. Express* **28**(7), 9713-9727(2020).

[2] Zhong J. et al. Observation of optical vortex knots and links associated with topological charge. *Opt. Express* **29**(23), 38849-38857(2021).

[3] Berry M V, & Dennis M R. Knotted and linked phase singularities in monochromatic waves. *Proceedings of the Royal Society of London. Series A: Mathematical, Physical and Engineering Sciences* **457**(2013), 2251-2263 (2001).

[4] Dennis, M. R., King, R. P., Jack, B., O’holleran, K., & Padgett, M. J. Isolated optical vortex knots. *Nat. Phys.* **6**(2), 118-121 (2010).

[5] Lu, X., Wang, Z., Zhao, C., Zhan, Q., & Cai, Y. Four-dimensional experimental characterization of partially coherent light using incoherent modal decomposition. *Nanophotonics* **12**(17), 3463-3470 (2023).

[6] Wang, Z., et al. Coherence phase spectrum analyzer for randomly fluctuated fractional vortex beam. *Photonics Res.* **12**(1), 33-39 (2024).

**Supplementary Figures**

**1. Numerical results of incoherent Hopf links.**


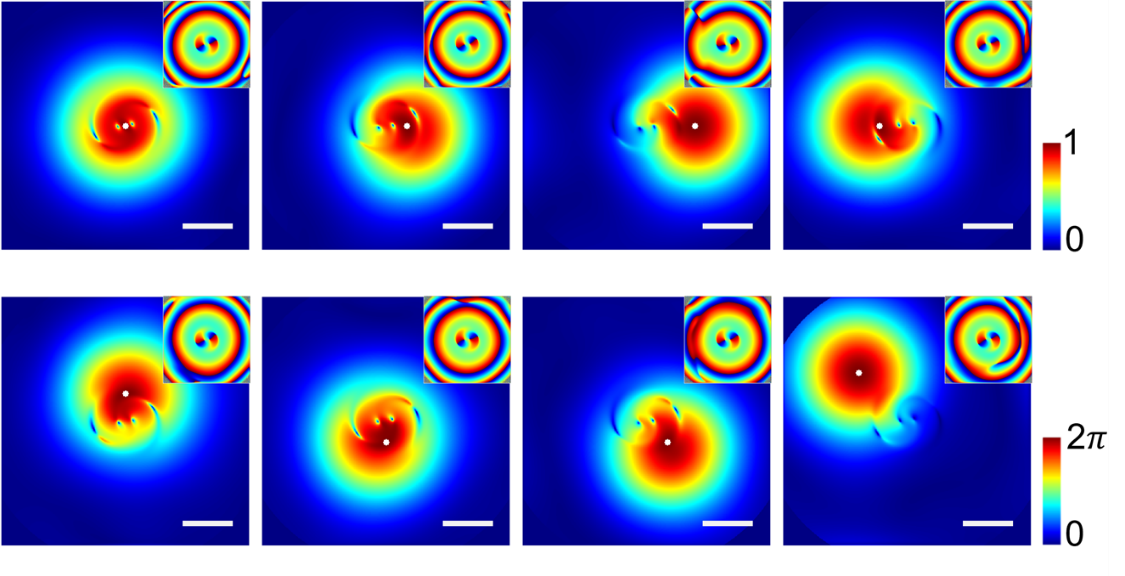


**Fig. S1 Numerical results of the phase and amplitude of the coherence function with incoherent topological structure at *z*=100 mm.** The phase structure (insets) remains nearly unchanged when choosing different reference points. White dots indicate reference points. The scale bars represent 0.6 mm.

**2.** **Numerical and experimental results of the incoherent light fields composed of speckles.**

**
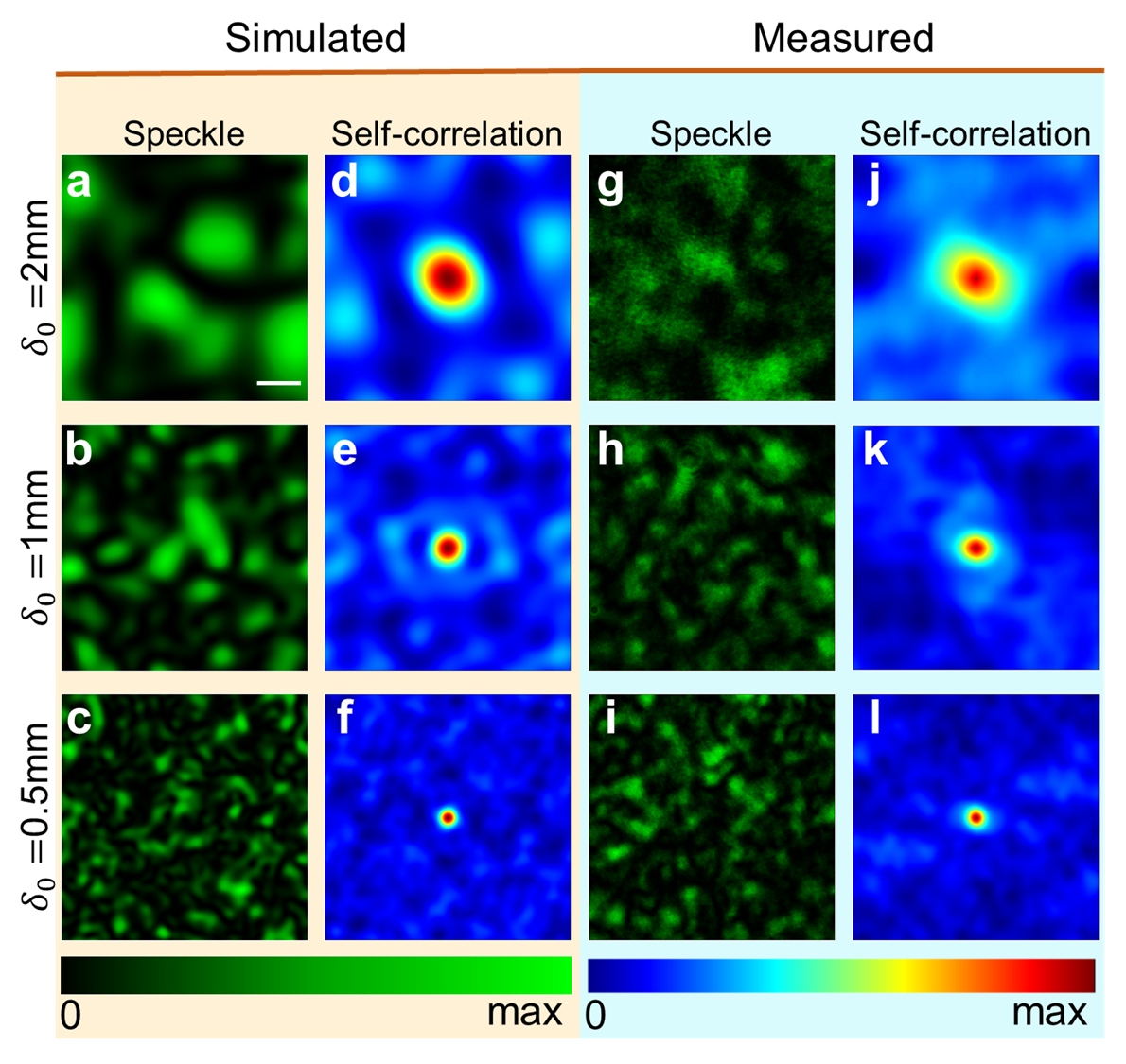
**

**Fig. S2 Numerical and experimental results of intensities and autocorrelations for incoherent light sources composed of speckles (before hologram).** **a-c, g-i**, Numerical and experimental intensities of speckles of different sizes. **d-f, j-l**, Corresponding autocorrelation functions with the size of Gaussian spot indicates degree of coherence or coherence length. The scale bars represent 2 mm.

**3. Numerical and experimental results of incoherent Hopf links and Trefoil knots.**

**
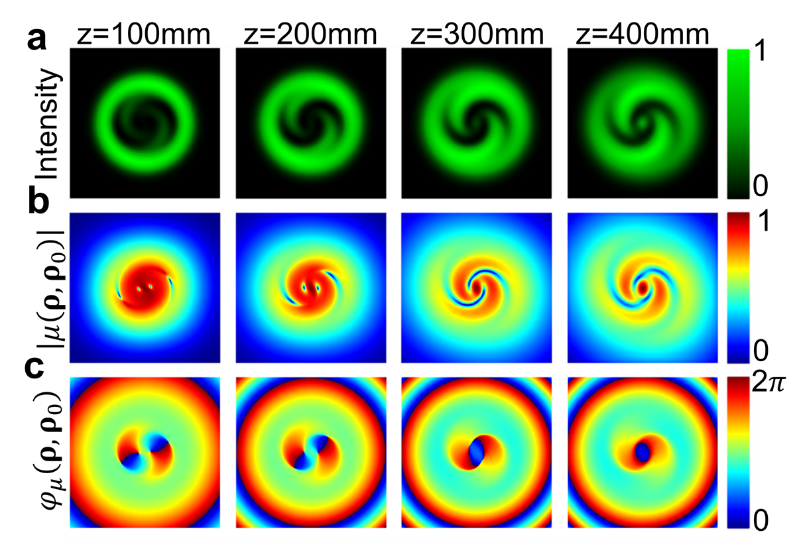
**

**Fig. S3 Numerical results of incoherent Hopf links with a light source of medium coherence during propagation.**

**
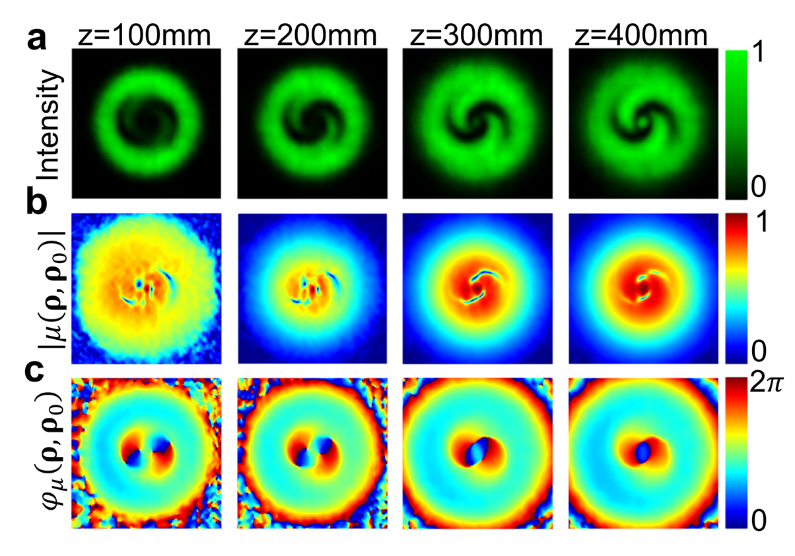
**

**Fig. S4 Experimental results of incoherent Hopf links with a light source of medium coherence during propagation.**

**
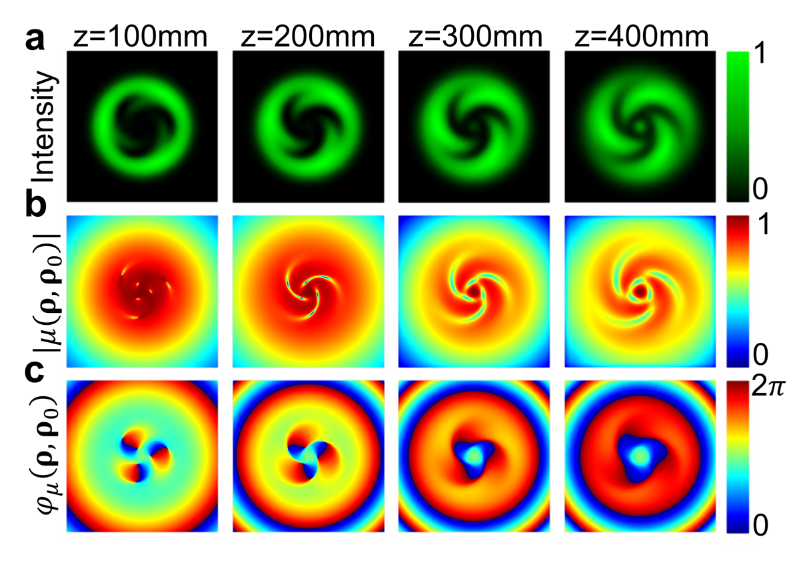
**

**Fig. S5 Numerical results of** **incoherent Trefoil knots with a light source of medium coherence during propagation.**

**
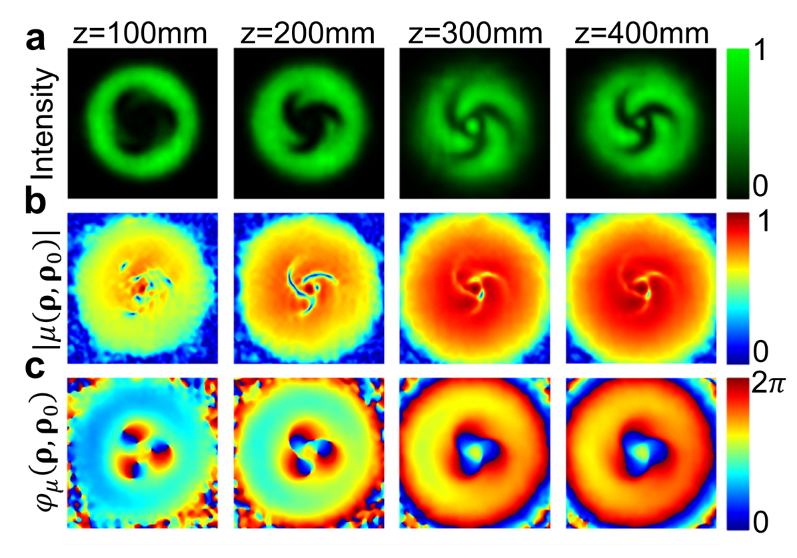
**

**Fig. S6 Experimental results of incoherent Trefoil knots with a light source of medium coherence during propagation.**

**
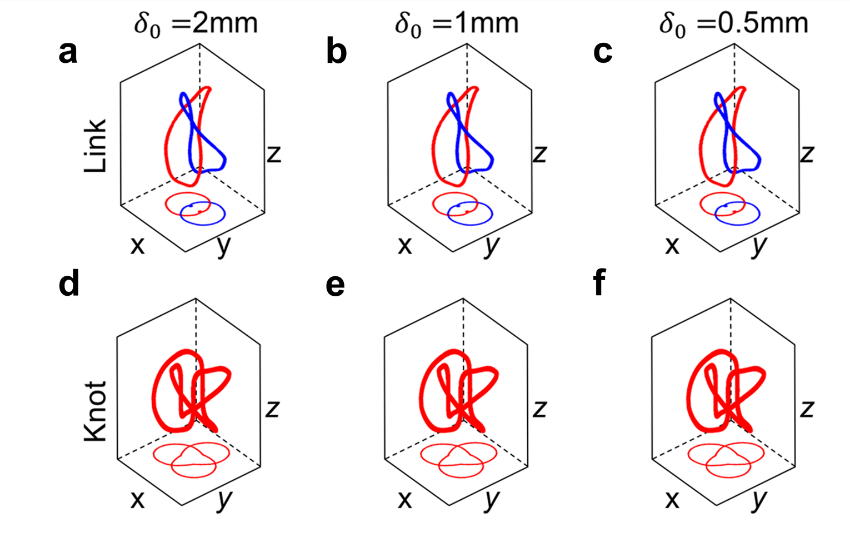
**

**Fig. S7** **Numerical results of 3D incoherent links and knots formed in incoherent light fields.** **a-c**, Incoherent Hopf links. **d-f**, Incoherent Trefoil knots.

**
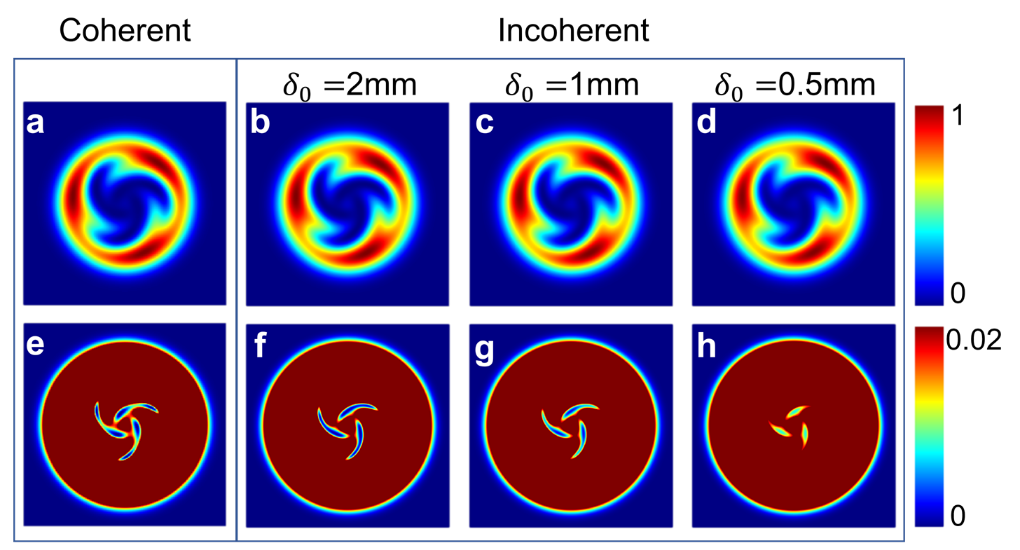
**

**Fig. S8 Numerical intensities of coherent and incoherent (different coherence lengths) Trefoil knots at *z*=200mm.**


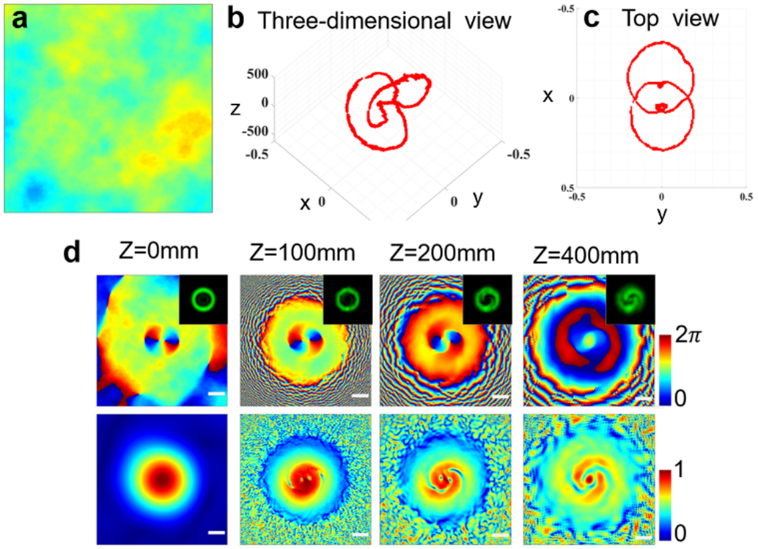


**Fig. S9 Numerical results of incoherent links with phase perturbation. a** represents the phase perturbation screen**. b** and **c** represent different views of the coherence link. d represents the coherence function results at different propagation distances. The scale bars represent 0.6 mm.


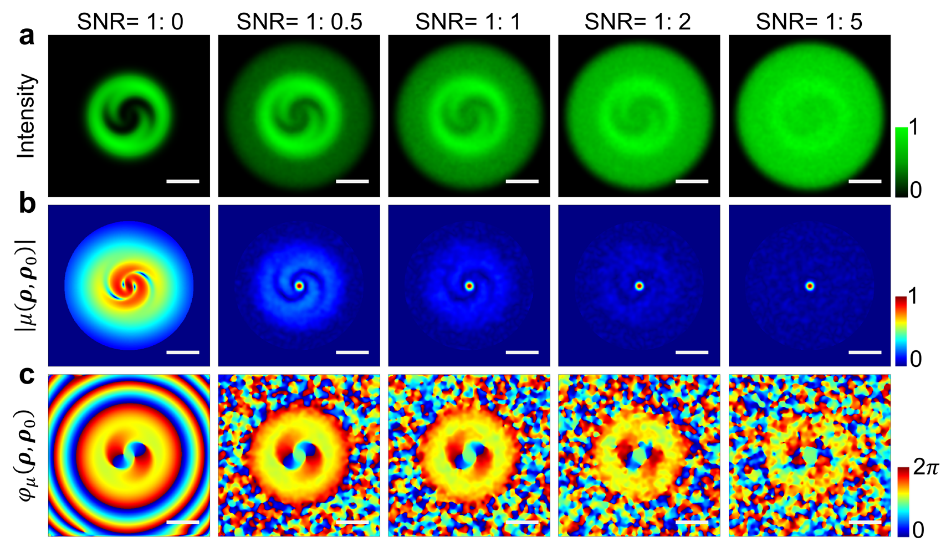


**Fig. S10 Numerical results of coherent singularities with varying intensity noise. a-c** are numerical results of the beam intensity, and the amplitude and phase of coherence function at propagation distance of *z* =200 mm. The scale bars represent 0.6 mm.


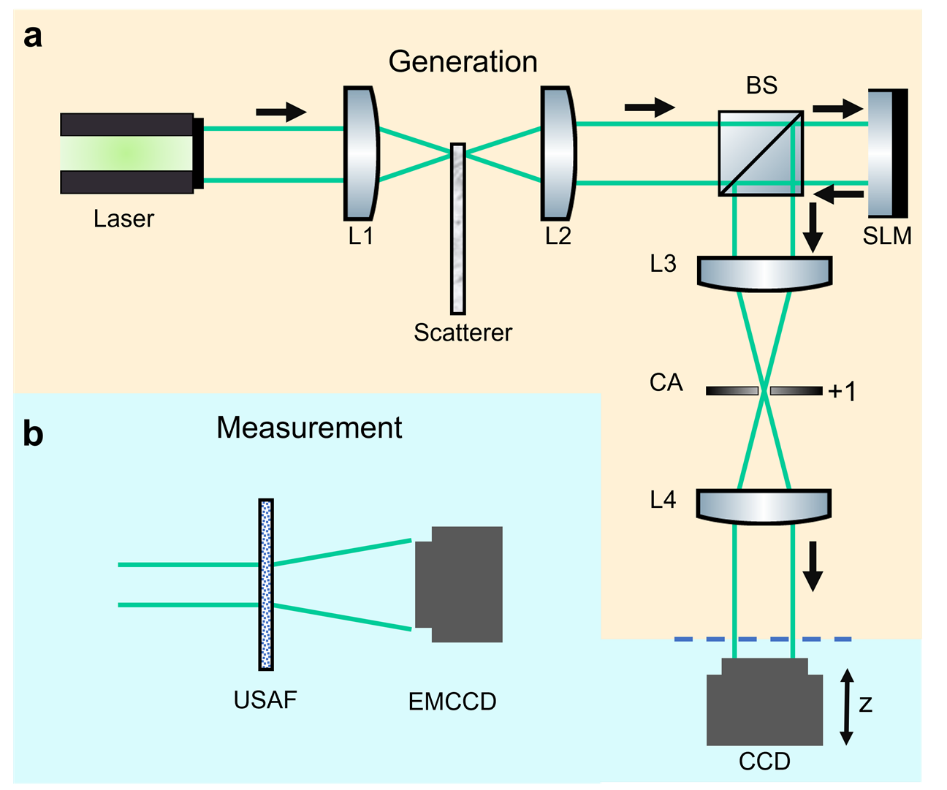


**Fig. S11 Schematic illustration of the experimental setup employed. a,** Experimental setup for the generation of coherence singularities and corresponding incoherent links and knots. BE, beam expander; L1, L2, L3, and L4, thin lenses; BS, beam splitter; SLM, spatial light modulator; CCD, charge-coupled detector (ECO445). **b**, Experimental setup for measuring coherence structures. USAF (1951USAF resolution test chart) acts as an object; EMCCD, electron multiplying CCD.
